# Siderophore-Based Noninvasive Differentiation of Aspergillus fumigatus Colonization and Invasion in Pulmonary Aspergillosis

**DOI:** 10.1128/spectrum.04068-22

**Published:** 2023-01-31

**Authors:** Dominika Luptáková, Rutuja H. Patil, Radim Dobiáš, David A. Stevens, Tomáš Pluháček, Andrea Palyzová, Marcela Káňová, Milan Navrátil, Zbyněk Vrba, Petr Hubáček, Vladimír Havlíček

**Affiliations:** a Institute of Microbiology of the Czech Academy of Sciences, Prague, Czechia; b Department of Analytical Chemistry, Palacký University, Olomouc, Czechia; c Department of Bacteriology and Mycology, Public Health Institute in Ostrava, Ostrava, Czechia; d Institute of Laboratory Medicine, Faculty of Medicine, University of Ostrava, Ostrava, Czechia; e California Institute for Medical Research, San Jose, California, USA; f Division of Infectious Diseases and Geographic Medicine, Stanford University School of Medicine, Stanford, California, USA; g Department of Anesthesiology and Intensive Care Medicine, University Hospital Ostrava, Ostrava, Czechia; h Institute of Physiology and Pathophysiology, Faculty of Medicine, University of Ostrava, Ostrava, Czechia; i Department of Intensive Medicine, Emergency Medicine and Forensic Studies, University of Ostrava, Ostrava, Czechia; j Department of Hematooncology, University Hospital Ostrava, Ostrava, Czechia; k Lung Department, Krnov Combined Medical Facility, Krnov, Czechia; l Department of Medical Microbiology, Charles University, Prague, Czechia; m Motol University Hospital, Prague, Czechia; Universidade de Sao Paulo

**Keywords:** *Aspergillus fumigatus*, colonization, invasive pulmonary aspergillosis, iron metabolism, mass spectrometry, noninvasive diagnosis, siderophore, urine analysis

## Abstract

Germination from conidia to hyphae and hyphal propagation of Aspergillus fumigatus are the key pathogenic steps in the development of invasive pulmonary aspergillosis (IPA). By applying *in vitro* observations in a clinical study of 13 patients diagnosed with probable IPA, here, we show that the transition from colonization to the A. fumigatus invasive stage is accompanied by the secretion of triacetylfusarinine C (TafC), triacetylfusarinine B (TafB), and ferricrocin (Fc) siderophores into urine, with strikingly better sensitivity performance than serum sampling. The best-performing index, the TafC/creatinine index, with a median value of 17.2, provided 92.3% detection sensitivity (95% confidence interval [CI], 64.0 to 99.8%) and 100% specificity (95% CI, 84.6 to 100%), i.e., substantially better than the corresponding indications provided by galactomannan (GM) and β-d-glucan (BDG) serology. For the same patient cohort, the serum GM and BDG sensitivities were 46.2 and 76.9%, respectively, and their specificities were 86.4 and 63.6%, respectively. The time-dependent specific appearance of siderophores in the host’s urine represents an impactful clinical diagnostic advantage in the early discrimination of invasive aspergillosis from colonization. A favorable concentration of TafC in a clinical specimen distant from a deep infection site enables the noninvasive sampling of patients suffering from IPA.

**IMPORTANCE** The importance of this research lies in the demonstration that siderophore analysis can distinguish between asymptomatic colonization and invasive pulmonary aspergillosis. We found clear associations between phases of fungal development, from conidial germination to the proliferative stage of invasive aspergillosis, and changes in secondary metabolite secretion. The critical extracellular fungal metabolites triacetylfusarinines C and B are produced during the polarized germination or postpolarized growth phase and reflect the morphological status of the proliferating pathogen. False positivity in Aspergillus diagnostics is minimized as mammalian cells do not synthesize Aspergillus siderophore or mycotoxin molecules.

## INTRODUCTION

Aspergillus fumigatus, a ubiquitous saprophytic mold with airborne conidia, is an opportunistic fungal pathogen that may cause severe and life-threatening human infections depending on the host’s immune status. A human typically inhales 10^3^ to 10^4^ conidia daily, which, due to their small size (<4 μm), can reach the alveoli and establish a core of infection (1, 2). Alveolar macrophages and epithelial cells in healthy individuals are highly effective in eliminating conidia. However, in immunosuppressed patients (with hematological malignancies, neutropenia, or hematopoietic stem cell or solid-organ transplants), conidia can germinate and subsequently cause invasive pulmonary aspergillosis (IPA), considered the most devastating type of Aspergillus-related pulmonary infection (3, 4). Globally, IPA affects approximately 200,000 patients annually, with a mortality rate of 30 to 95% (2). It is also increasingly being reported in patients with underlying respiratory diseases such as severe asthma, chronic obstructive pulmonary disease (COPD), and superinfection with influenza or coronavirus disease 2019 (COVID-19), with almost 30% morbidity in some instances (3, 5). These outcomes highlight the importance of understanding the A. fumigatus invasion mechanism, which could help in the establishment of early and specific diagnostic platforms.

A promising diagnostic approach is to exploit a survival system that A. fumigatus has evolved, which plays a key role in its nutrient acquisition during successful host invasion and pathogenicity. Iron is an essential trace element for fungal growth and development (6, 7), but the availability of usable iron is limited, so adaptation to iron starvation, with the concurrent detoxifying elimination of excessive iron uptake (8), is crucial for virulence (9). The system involves the secretion of low-molecular-mass, high-affinity, ferric ion-specific chelators called siderophores that facilitate iron storage and acquisition in conjunction with specific siderophore-iron transporters (8, 10). Siderophore production is tightly regulated during fungal germination and subsequent developmental processes. The distinct roles of intracellular ferricrocin (Fc), hydroxyferricrocin (Hfc), extracellular fusarinine C (FsC), and triacetylfusarinine C (TafC) siderophores during A. fumigatus infection maintain iron homeostasis, conidial and hyphal iron storage, hyphal iron trafficking, reproductive and developmental processes, and oxidative stress resistance (6, 8, 10, 11). Moreover, siderophores have been shown to serve as specific biomarkers of microbial infections (12). Additionally, A. fumigatus produces mycotoxins, including (among others) immunosuppressive gliotoxin (Gtx) (13), fumagillin, and fumitremorgin A (14, 15). Although mycotoxins are less specific Aspergillus biomarkers, their detection could be helpful in the diagnosis of IPA in neutropenic patients who have negative serum galactomannan (GM) assay results (16).

The early, sensitive, and specific diagnosis of Aspergillus-caused infections remains challenging, and the distinction between A. fumigatus colonization and invasion of the respiratory tract is also unclear. Current IPA diagnostics rely on the observation of pulmonary nodules by computed tomography (CT), serological testing for GM and 1,3-β-d-glucan (BDG), Aspergillus DNA detection in different specimens by quantitative PCR (qPCR)-mediated amplification, and various kinds of mycological (including microscopic, histological, and culture) examinations (4, 17, 18). In experimental studies, the extent of Aspergillus infection has been visualized by mass spectrometry (MS) imaging of Fc siderophore (19). TafC and Fc have also been detected in rat urine as early as 4 h after infection (20). TafC has promising utility as a urine (21), serum (22), and bronchoalveolar lavage fluid (BALF) (23) biomarker of IPA in humans.

Here, we demonstrate the potential utility of infection metallomics (24) for the clinical diagnosis of IPA based on the noninvasive detection in urine of the intra- and extracellular siderophore TafC, its hydrolytic product triacetylfusarinine B (TafB) (11), Fc, and a secondary metabolite, Gtx. We present clear links between germination or infection phase-dependent siderophore production and observations in critically ill patients. The active secretion of pathogen’s virulence factors (9) into the blood is a putative marker of the proliferation and angioinvasion of Aspergillus (12). This is the first report of the presence of TafB and Gtx in human urine samples and their potential value, together with TafC, for monitoring A. fumigatus invasion and the early, accurate diagnosis of IPA.

## RESULTS

### Siderophore profiles are growth phase dependent.

**FIG 1:**
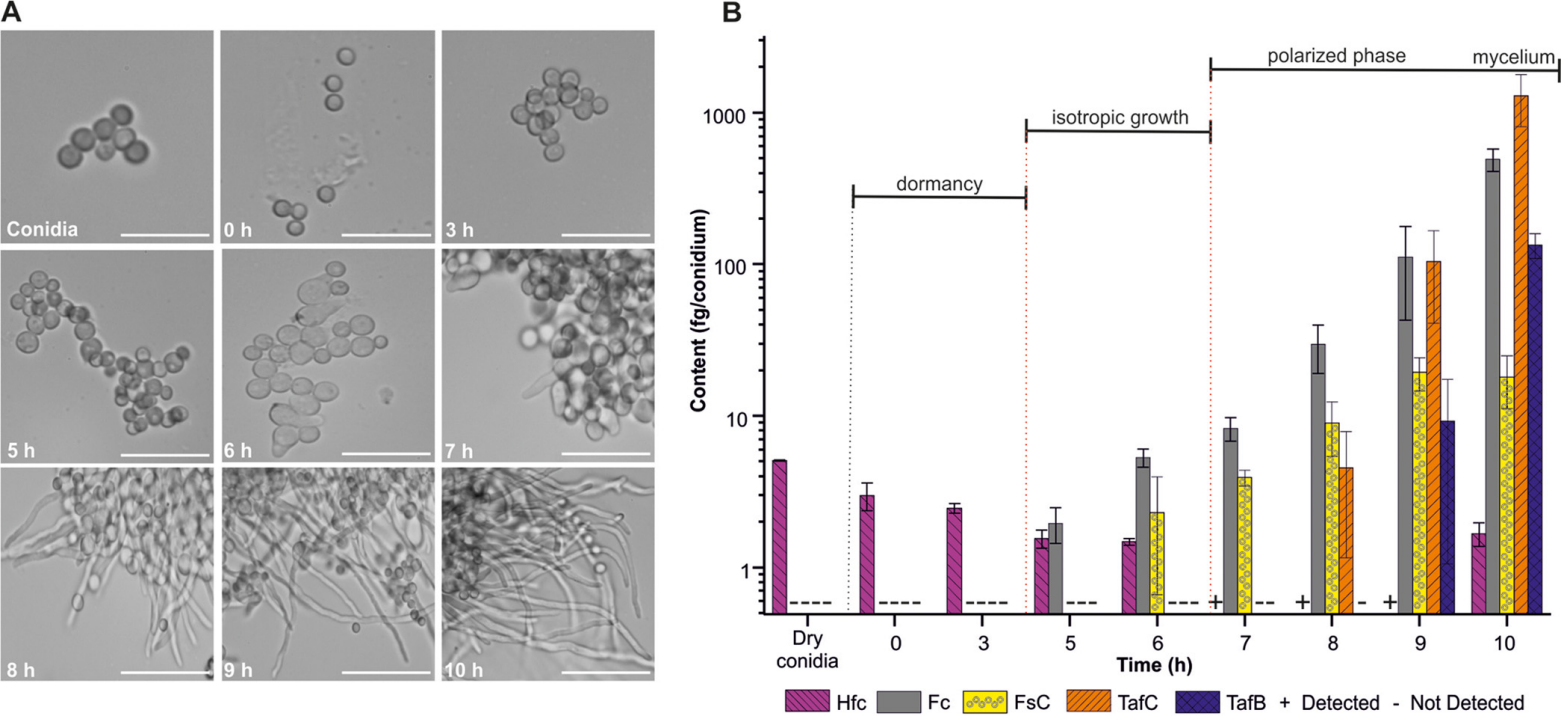
Time course of A. fumigatus conidial germination in flask cultures. (A) Bright-field microscopic images of A. fumigatus conidia illustrating their germination phases, dormancy (0 to 3 h postinoculation) (top row), isotropic growth (3 to 6 h) (middle row), and polarized growth (6 to 10 h) (bottom row). Bars, 10 μm. (B) Levels of intra- and extracellular siderophores recorded during germination of A. fumigatus conidia. Bar graphs represent the averages from four biological replicates. Data are presented as means ± standard deviations. Hfc, hydroxyferricrocin; Fc, ferricrocin; FsC, fusarinine C; TafC, triacetylfusarinine C; TafB, triacetylfusarinine B.

To quantify the production of siderophores during A. fumigatus growth, we measured their concentrations (shown as means ± standard deviations [SDs] in this section) at key stages during the course of conidial germination (Fig. 1), involving incubation of conidia for 72 h at 37°C in iron-limited medium with glucose as a carbon source, by high-performance liquid chromatography-mass spectrometry (HPLC-MS). During the first 3 h of incubation, no morphological changes were observed in conidia, indicating conidial dormancy. Isotropic growth was observed between 3 and 6 h after inoculation. At 6 h, most conidia began swelling and doubled in size from their initial, dormancy state. Polarized growth started from 6 h postinoculation. By 8 h, most conidia had germ tubes growing from one side of the cell (Fig. 1A).

**FIG 2:**
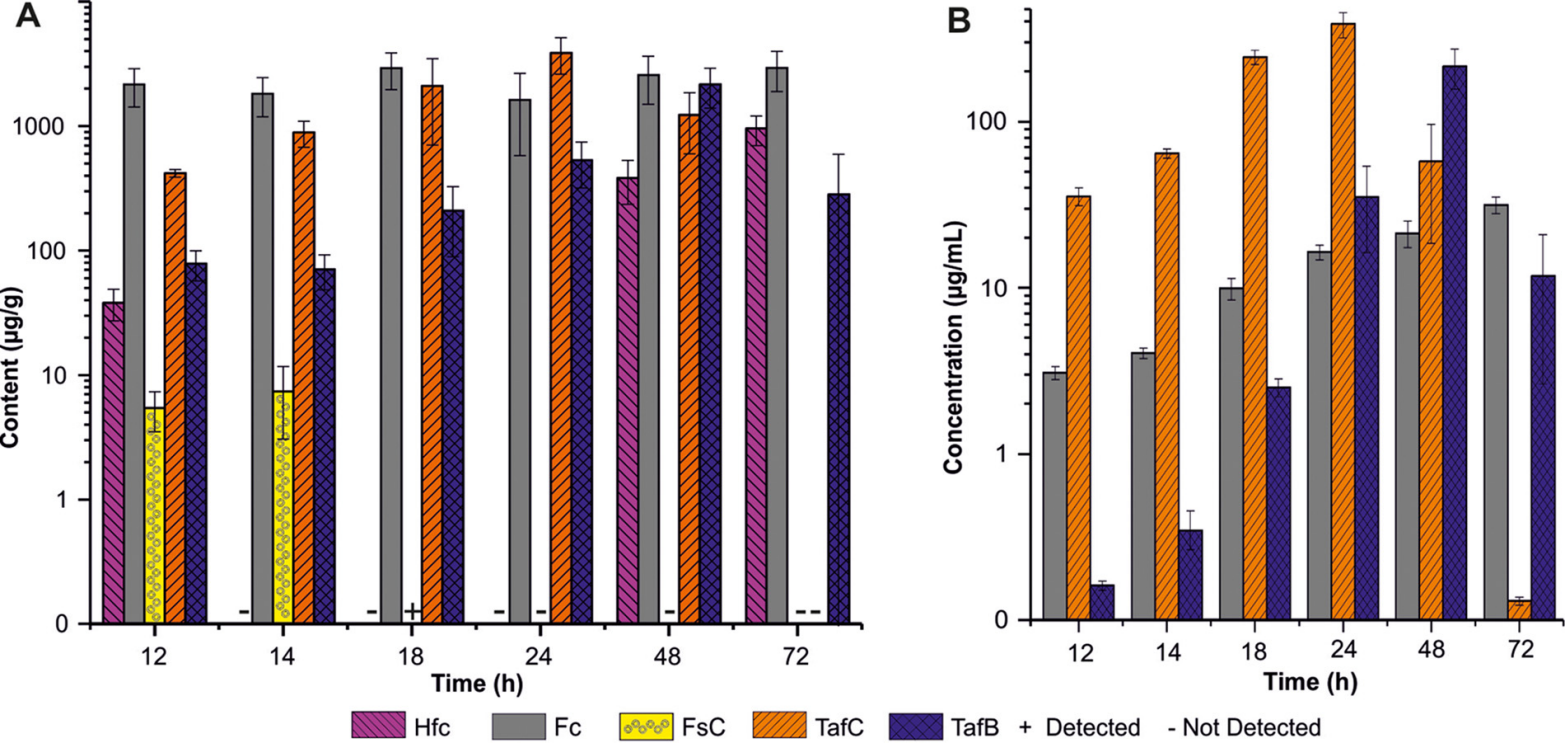
Quantitative profiling of A. fumigatus siderophores after the completion of germination in the residual fungal mass (A) and the supernatant (B) from 12 to 72 h postinoculation. Bar graphs represent the averages from four biological replicates. Data are presented as means ± standard deviations. Hfc, hydroxyferricrocin; Fc, ferricrocin; FsC, fusarinine C; TafC, triacetylfusarinine C; TafB, triacetylfusarinine B.

We detected clear germination phase-dependent variations in the abundances of siderophores involved in iron homeostasis produced by A. fumigatus (Fig. 1 and 2). Intracellular Hfc, responsible for conidial iron storage (25), was detected mainly in dormant conidia, with a maximum content of 5.07 ± 0.03 fg/conidium (Fig. 1B). In the late stage of cultivation (declining phase), with the transition to the stationary or autolytic phase, the Hfc level in the residual fungal mass rose to 955 ± 255 μg/g, while no Hfc was detected in the supernatant (Fig. 2). The reappearance of intracellular Hfc after 48 h (Fig. 2A) could be a marker of new sporulation at the air-glass interface, which cannot be eliminated in shaken cultures (25).

The concentration of Fc, an intracellular and partially extracellular siderophore essential for conidial formation and germination (10), gradually increased during isotropic growth. At 5 h postinoculation, the Fc content was 1.95 ± 0.52 fg/conidium (Fig. 1B). From 12 to 72 h, Fc accumulated in mycelia from 1,618 ± 1,038 to 2,944 ± 1,050 μg/g (Fig. 2A), highlighting the enormous iron requirement for mycelium maturation. Concurrently, the Fc concentration gradually increased to 31.51 ± 3.62 μg/mL in the culture supernatant (Fig. 2B).

TafC secretion was detected from 8 h postinoculation during the polarized growth of conidia, with a rapid increase from 4.53 ± 3.37 to 1,289 ± 483 fg/conidium (Fig. 1B). TafC accumulated in the supernatant over 24 h, reaching a maximum of 386 ± 68 μg/mL (Fig. 2B). In conidia, FsC (the precursor of TafC) was detected from 6 h postinoculation (at 2.30 ± 1.64 fg/conidium) in the isotropic phase, while TafB, a hydrolytic product of TafC, was observed in the late polarized stage, at 9 h postinoculation (at 9.19 ± 8.14 fg/conidium) (Fig. 2B). Interestingly, TafB, which is produced by opening one ester bond of TafC (11), became more abundant than TafC in the stationary phase (48 h postinoculation) of fungal growth in the supernatant (Fig. 2B).

### The content of A. fumigatus mycotoxins in the residual fungal mass is constant.

Depending on the growth conditions, A. fumigatus responds to stress factors by producing multiple mycotoxins. In our *in vitro* experiment, we quantified the tryptophan-derived peptidyl alkaloids fumiquinazoline C (14.57 ± 8.49 to 38.90 ± 2.4 fg/conidium), fumiquinazoline D (6.24 ± 0.33 to 20.61 ± 2.88 fg/conidium), 3-hydroxy-fumiquinazoline A (from the limit of quantitation [LOQ] to 12.97 ± 0.80 fg/conidium), tryptoquivaline F/J (5.58 ± 0.28 to 9.23 ± 1.16 fg/conidium), and the ergot alkaloid fumigaclavine A (from the LOQ to 71.39 ± 26.32 fg/conidium) during germination (see Fig. S1 in the supplemental material). The content of mycotoxins was constant in residual fungal mass, and no mycotoxins were detected in the supernatant. Notably, we did not see any Gtx production *in vitro*, which contradicts multiple Gtx findings in patients with invasive pulmonary aspergillosis (16).

### Fungal secondary metabolite detection in urine provides a noninvasive diagnosis of IPA.

**FIG 3:**
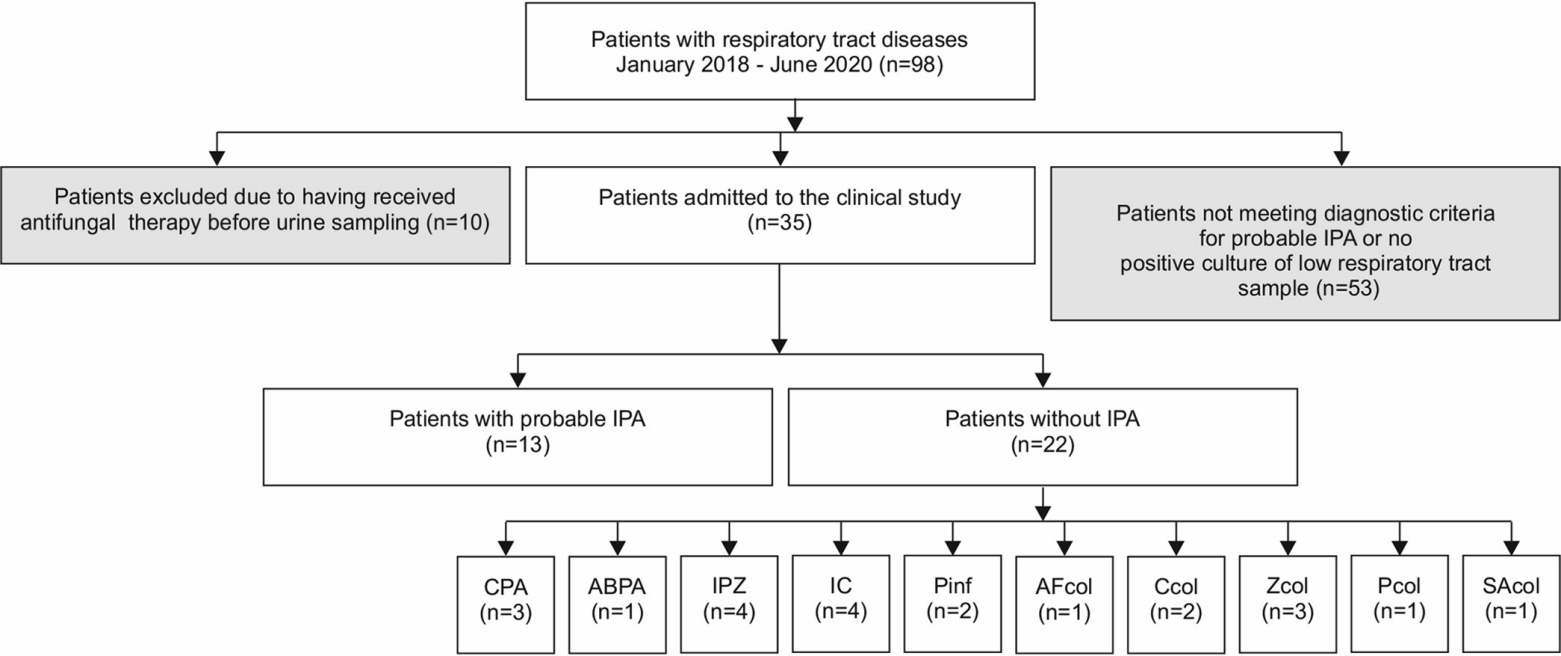
Classification of patients in the clinical study. IPA, invasive pulmonary aspergillosis; CPA, chronic pulmonary aspergillosis; ABPA, allergic bronchopulmonary aspergillosis; IPZ, invasive pulmonary zygomycosis; IC, invasive candidiasis; Pinf, Pseudomonas aeruginosa infection; AFcol, A. fumigatus colonization; Ccol, *Candida* species colonization; Zcol, zygomycete colonization; Pcol, P. aeruginosa colonization; SAcol, Scedosporium apiospermum colonization; n, number of patients.

In the 3-year observational, retrospective, noninterventional clinical study, 35 patients were enrolled (Fig. 3), of whom 13 patients, with a 12:1 male/female ratio (see the patient cohort characteristics in Materials and Methods), were diagnosed with probable IPA (26, 27). The clinical specimens in all 13 cases were not obtained from primarily sterile material. Of note, 12 of the 13 patients responded, when antifungal therapy was given, by clinical and laboratory examinations. The diagnosis of IPA included underlying risk factors and diseases, most often including the administration of immunosuppressants (*n* = 4); neutropenia and influenza (*n* = 3); and COPD, bronchopneumonia, and multiple myeloma (*n* = 3) (Table 1). To visualize possible biomarker correlations, the data in Table 1 are displayed in a graphic form in Fig. S2. The control patient cohort is represented by 22 patients diagnosed with non-IPA infections, including chronic pulmonary aspergillosis (CPA), invasive pulmonary mucormycosis, invasive candidiasis, and colonization with A. fumigatus, among others (Fig. 3). Other control patients suffered from polytrauma (*n* = 5) and COPD (*n* = 3) (Table S1).

**TABLE 1:** Results of analyses of urine samples from immunocompromised and immunocompetent patients for the noninvasive detection of IPA^*a*^

Underlying disease	Risk factor(s)		Infection metallomics (mean value ± SD)^*b*^			Conventional biomarker		Death
		uGtx/Crea index	uFc/Crea index	uTafC/Crea index	uTafB/Crea index	sGM (ODI)	sBDG (pg/mL)	
Multiple myeloma	Neutropenia, IST	15.8 ± 1.0	Det	17.2 ± 0.2	ND	1.56	501	No
Non-Hodgkin lymphoma	Neutropenia, IST	Det	ND	32.1 ± 0.4	ND	3.14	115	No
Multiple myeloma	Neutropenia, IST	ND	ND	Det	ND	0.17	ND	Yes
Bronchopneumonia	Flu (H1N1)	85.2 ± 3.7	8.3 ± 0.7	82.9 ± 1.5	5.4 ± 0.1	5.9	276	Yes
COPD	Steroids	ND	11.9 ± 2.7	35.7 ± 0.4	11.5 ± 0.4	0.89	>523	Yes
COPD	Flu (H1N1)	ND	ND	Det	ND	0.1	161	No
DM II	Ketoacidosis	33.8 ± 1.4	4.2 ± 0.5	2.3 ± 0.1	ND	0.26	155	No
Bronchopneumonia	Flu (H1N1), steroids	67.9 ± 6.6	40.6 ± 2.2	3,117.4 ± 78.2	59.6 ± 3.0	7.9	>523	Yes
Liver transplant	Steroids, IST	ND	ND	27.6 ± 0.3	4.7 ± 0.3	0.06	221	No
Bronchopneumonia	Steroids	ND	ND	12.9 ± 0.3	ND	0.56	>523	No
Polytrauma		ND	ND	ND	ND	0.24	75	No
COPD	DC	596.3 ± 13.1	505.1 ± 8.0	1,071.1 ± 5.6	250.4 ± 2.2	0.126	>523	No
Burns		ND	ND	Det	ND	0.121	ND	No

a All cases (13 in total) were classified as “probable aspergillosis” (26, 27). uFc, ferricrocin in urine; uTafC, triacetylfusarinine C in urine; uTafB, triacetylfusarinine B in urine; uGtx, gliotoxin in urine; sGM, galactomannan in serum; sBDG, β-d-glucan in serum; ND, not detected; Det, detected; Crea, creatinine; ODI, optical density index; COPD, chronic obstructive pulmonary disease; DM II, diabetes mellitus type II; IST, immunosuppressants; DC, decompensated cirrhosis; Flu, influenza.

b For creatinine indexing, see reference 21.

Applying our infection metallomics strategy (24), the characteristic A. fumigatus siderophores identified from the *in vitro* study (Movie S1) were screened for in all patient urine and serum samples. Fc, TafC, TafB, and Gtx were the main secondary metabolites detected in IPA patients (Tables 1 and 2; Table S2). Importantly, none of these molecules were detected in the controls (Table S1). The biological variability in patients with diverse renal functions was partly compensated for by creatinine indexing (Tables 1 and 2), originally used for TafC quantitation by Hoenigl et al. (21). The TafC/creatinine ratio (“index”) in urine (median value, 17.2 [interquartile range {IQR}, 1.5 to 59.3]) provided 92.3% detection sensitivity (95% confidence interval [CI], 64.0 to 99.8%) for probable IPA. Lower sensitivity was offered by the Fc/creatinine index (46.2% [95% CI, 19.2 to 74.9%]), the Gtx/creatinine index (46.2% [95% CI, 19.2 to 74.9%]), and the TafB/creatinine index (38.5% [95% CI, 13.9 to 68.4%]), with median values of 0.9 (IQR, 0.9 to 10.1), 2.7 (IQR, 2.7 to 50.9), and 0.6 (IQR, 0.6 to 8.5), respectively.

**TABLE 2:** LOD, sensitivity, and specificity values for analyses of urine samples from immunocompromised and immunocompetent patients for the noninvasive detection of IPA^*a*^

Biomarker	LOD threshold	Sensitivity (%) (95% CI)	Specificity (%) (95% CI)
Infection metallomics^*b*^			
uGtx/Crea index	≥2.7 ng/mL	46.2 (19.2–74.9)	100 (84.6–100)
uFc/Crea index	≥0.9 ng/ml	46.2 (19.2–74.9)	100 (84.6–100)
uTafC/Crea index	≥0.6 ng/mL	92.3 (64.0–99.8)	100 (84.6–100)
uTafB/Crea index	≥0.6 ng/mL	38.5 (13.9–68.4)	100 (84.6–100)
Conventional approaches			
sGM	≥0.5 ODI	46.2 (19.2–74.9)	86.4 (65.1–97.1)
sBDG	≥80 pg/mL	76.9 (46.2–95.0)	63.6 (40.7–82.8)

a All cases (13 in total) were classified as “probable aspergillosis” (26, 27). uFc, ferricrocin in urine; uTafC, triacetylfusarinine C in urine; uTafB, triacetylfusarinine B in urine; uGtx, gliotoxin in urine; sGM, galactomannan in serum; sBDG, β-d-glucan in serum; Crea, creatinine; CI, confidence interval.

b For creatinine indexing, see reference 21.

Serum analysis of fungal secondary metabolites showed poor detection sensitivities (Table S2), at 23.1% (95% CI, 5.0 to 53.8%) for Gtx and Fc and 15.4% (95% CI, 1.9 to 45.4%) for TafC (22).

HPLC-MS provided 100% specificity for all siderophores and Gtx (95% CI, 84.6 to 100%) for both the urine and serum samples. We also detected strong positive correlations in the levels of individual urine biomarkers and standard clinical diagnostic serological tests, i.e., between TafC and TafB (*r *= 0.841; *P *= 0.0005), TafC and Fc (*r *= 0.769; *P *= 0.0037), Fc and TafB (*r *= 0.800; *P *= 0.0020), Fc and Gtx (*r *= 0.766; *P *= 0.0031), and TafC and BDG (*r *= 0.743; *P *= 0.0053). Moderate positive correlations were found between TafC and Gtx (*r *= 0.691; *P *= 0.0118), TafB and BDG (*r *= 0.679; *P *= 0.0130), and Fc and BDG (*r *= 0.679; *P *= 0.0138). No significant correlation was detected between serum GM and either secreted urine biomarkers or BDG (Table S3). Notably, compared to urine analysis, GM and BDG serology provided far lower detection sensitivities for this patient cohort, at 46.2% (95% CI, 19.2 to 74.9%) and 76.9% (95% CI, 46.2 to 95.0%), respectively, as well as lower specificities, at 86.4% (95% CI, 65.1 to 97.1%) and 63.6% (95% CI, 40.7 to 82.8%), respectively.

## DISCUSSION

**FIG 4:**
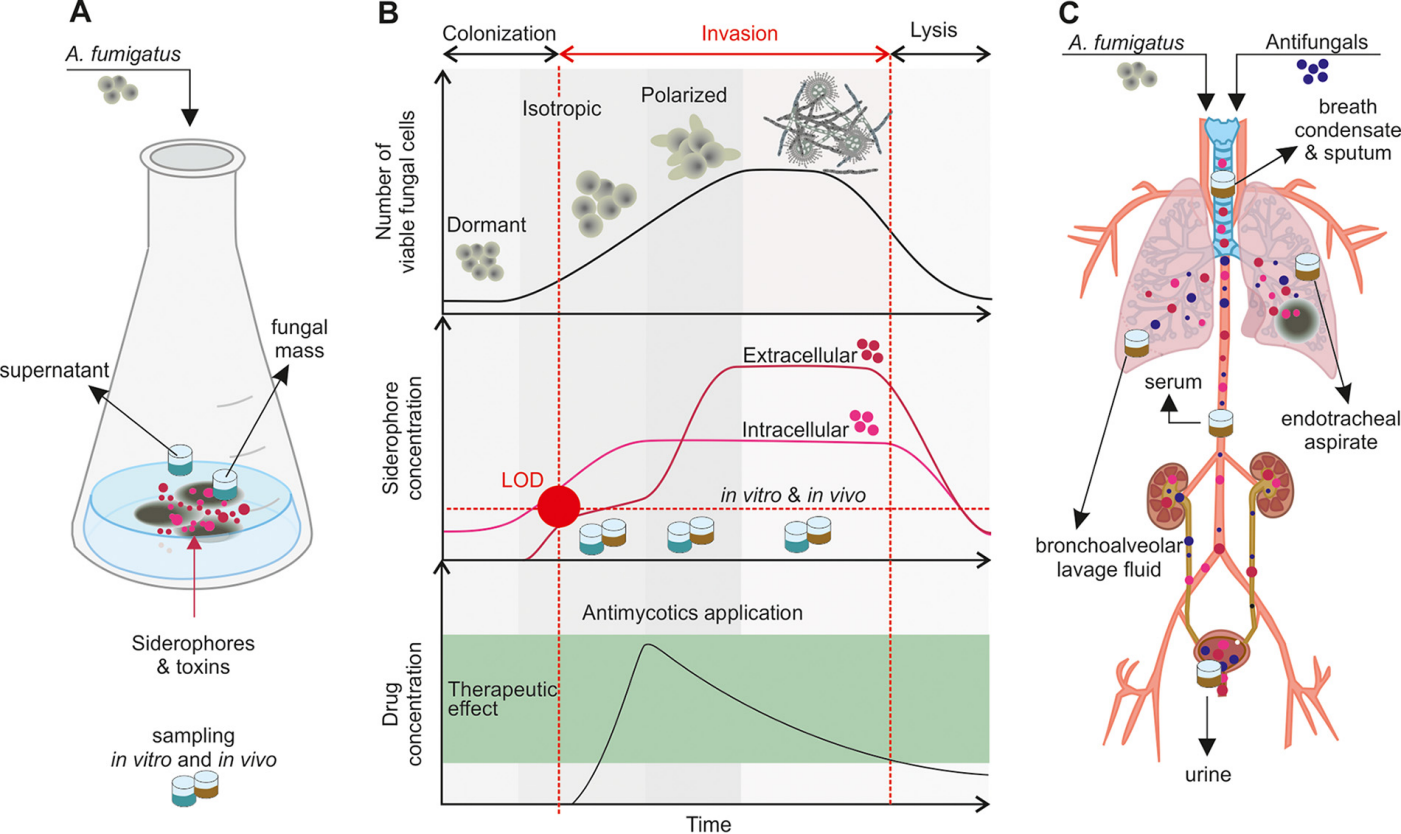
Characterization of the *in vitro* fungal growth-dependent siderophore profile defining the transition between A. fumigatus colonization and invasion in critically ill patients. (A) A. fumigatus cultivated *in vitro* produces virulence factors (siderophores and secondary metabolites) that are either stored in conidia and hyphae or secreted into the cultivation medium. (B) Conidial germination involves progression through dormancy, isotropic growth, and the polarized growth phase to the production of hyphae and the formation of mycelium. Under iron-limited conditions, the fungus secretes iron chelators: first intracellular siderophores and then extracellular siderophores. If the growth conditions are modified, e.g., by the application of antifungal compounds, lysis of the fungal hyphae may occur, followed by a decrease in virulence factor production. (C) A. fumigatus entering a human host competes for scarce nutrients and scavenges iron using siderophores, which are spread in the infected tissue due to hyphal angioinvasion. If antifungal treatment has started, changes in the siderophore concentration profile may reflect the therapeutic effect. LOD, limit of detection.

The level of TafC retention in the blood is low, and its renal clearance is efficient, as documented in rats by positron emission tomography (28). The fungal growth-dependent production of siderophores that mammalian hosts do not synthesize could enable the differentiation of colonization and invasion (infection) by A. fumigatus in critically ill patients. Our central hypothesis is that the transition to A. fumigatus invasion is accompanied by the appearance of fungal siderophores, as a response to an increased need for iron, in body fluids as a result of molecular leakage (12) (Fig. 4). Thus, their appearance in a host, defined by the limit of detection (LOD) of the infection metallomics approach (24), provides early, sensitive, and specific noninvasive detection of A. fumigatus-induced IPA in humans.

Depending on the host’s status (29), once a conidial site is established, with subsequent germination, tissue invasion, and angioinvasion, fungal virulence factors are disseminated to other organs via the bloodstream. The human body is a “continuous-flow system” in which A. fumigatus-excreted siderophores are distributed from the infection site(s) to various host matrices, including the circulation, from which they are filtered into the urine (Fig. 4C). Their concentrations may be affected by changes in the pathogen’s viability in response to host factors or antifungal treatment (Fig. 4B).

The results for *in vitro* fungal germination were applied to the diagnosis of invasive pulmonary aspergillosis *in vivo*. Conidial germination, induced by micronutrient stimuli (carbon and nitrogen), can be asynchronous (30). As a result, heterogeneity in cell populations was observed (Fig. 1). In agreement with results reported previously by Danion et al. (30), early germ tubes were formed at 6 h, and the germination of all remaining conidia was completed in 10 h (30). In general, A. fumigatus mycelia are grown from an appropriate morphotype (31) (conidial, isotropic growth, or polarized growth state) depending on strain origin, micronutrient availability, temperature, and pH (32). With the inoculum that we used in the *in vitro* model, specific siderophores secreted at each stage of A. fumigatus growth were measured by HPLC-MS-based infection metallomics. The detection of Hfc in dormant conidia proved its conidial character (25). Later, its presence in the residual fungal mass was correlated with the formation of asexual conidiophores resulting from a combination of isotropic and polarized cell growth *in vitro* leading to repeated conidial maturation and fungal mass expansion (33). Predominantly intracellular Fc marked the onset of isotropic growth, manifested by the swelling of conidia (34). The detection of an exponential increase in its contents, up to the constant level in the residual fungal mass, may reflect the accumulation of the iron needed to sustain hyphal growth and further conidiation. The data on Fc intracellular production during isotropic, polarized, and vegetative growth may be correlated with the corresponding transcripts detected by transcriptome sequencing (9, 30). Note that a gradual increase in the concentration of Fc, which is reportedly transcellular (35), was also detected in the supernatant. Alternatively, Fc could be released due to mechanical stress in a shaken flask culture (or phagocytosis *in vivo*).

In our *in vitro* study, the gradual increase in FsC secretion into the culture supernatant during isotropic growth was followed by the secretion of TafC and its hydrolytic derivative TafB (36) during the fungal transition to polarized growth. The TafB level subsequently increased to maximum levels, both intra- and extracellularly, at the expense of TafC. This inverse relationship of the abundances of TafC and TafB reflected the iron capture, release, or storage steps managed by Aspergillus. The decreasing TafC and TafB levels in the supernatant in the later course of the fungal growth cycle are illustrated in Fig. 2B.

FsC dominates in the cell during initial hyphal elongation (32) and represents the intracellular precursor of TafC (37). Once TafC amounts are sufficient and fungal need for iron is saturated, SreA- and HapX-mediated iron regulation (38, 39) starts to block gene expression involved in TafC synthesis via FsC. In parallel, TafC, as a main transporter of iron to the fungal cell, is enzymatically hydrolyzed to TafB (11, 36). The released iron can be used in cellular metabolic pathways or stored in ferricrocin (we see a constant intracellular Fc content) or in vacuoles (38) to help cell detoxification.

During angioinvasion, proliferating fungal hyphae invade the endothelial cell linings and pulmonary blood vessels by passing from their abluminal to their luminal side, which results in pulmonary hemorrhage and vascular thrombosis at the infection site. Apart from tissue infarction, fungal hyphae are fragmented and further circulated in the bloodstream (40). Circulating A. fumigatus hyphae thus disseminate and secrete secondary metabolites and virulence factors into the blood, followed by the renal filtration of these soluble products, allowing direct monitoring of the disease status (14, 41). Specifically, the occurrence of Fc in urine could be related to its extracellular character (11, 20). Notably, antifungal treatment might contribute to the release of fungal cell intracellular contents, thereby contributing to the abundance of Fc in urine.

The detection of mycotoxins, including fumigaclavine A, fumiquinazoline C, fumiquinazoline D, 3-hydroxy-fumiquinazoline A, and tryptoquivaline F/J, exclusively in conidia demonstrated the ability of A. fumigatus to adapt to environmental conditions (14). Note that tryptoquivaline F and tryptoquivaline J are isobaric.

Our work is the first report of TafB and Gtx secretion into human urine. Together with TafC (21) and Fc (20), which have already been used, we now have a diagnostic panel that may provide better predictability of IPA severity than current conventional approaches. Interestingly, the sensitivity of TafC in serum (2 of 13 patients with IPA) was surprisingly very poor (Table S4).

In conclusion, analysis of A. fumigatus growth phase-dependent siderophore production can discriminate between asymptomatic colonization and invasive infection. Urine is an ideal matrix for noninvasive and repeated sampling of critically ill patients, unless they have renal insufficiency. The excellent TafC/creatinine index in the urine, now provided by mass spectrometry, may serve as a basis for next-generation infection diagnostics. Note that aspergillosis can also be caused by Aspergillus species that do not produce TafC, such as Aspergillus terreus. For these rare aspergillosis cases, the TafC/creatinine index cannot be used for diagnostic purposes.

## MATERIALS AND METHODS

### Chemicals.

Standard ferrioxamine E (FoxE), Fc, and TafC ferriforms and Gtx were obtained from EMC Microcollections GmbH (Tübingen, Germany). Formic acid (FA) and fumigaclavine A were purchased from VWR Chemicals (Stříbrná Skalice, Czechia). Fumiquinazoline D was obtained from Spinchem (Plzen, Czechia). HPLC-grade methanol (MeOH), acetonitrile (ACN), and water were purchased from Honeywell (Seelze, Germany). Na_2_HPO_4_·12H_2_O, KH_2_PO_4_, NaCl, NH_4_Cl, glucose, MgSO_4_·7H_2_O, CaCl_2_·2H_2_O, ZnSO_4_·7H_2_O, MnSO_4_·H_2_O, CoCl_2_·6H_2_O, CuSO_4_·5H_2_O, and Na_2_MoO_4_ were obtained from LachNer Chemicals (Neratovice, Czechia). FeCl_3_·6H_2_O and Tween 80 were purchased from Lachema (Brno, Czechia).

### Microorganism.

A. fumigatus strain EI278 was isolated from a clinical sample received from the University Hospital in Motol (Prague, Czechia) and identified by phylogenetic analysis of the internal transcribed spacer 1 (ITS1) and ITS2 regions. Fungal cultures were grown in yeast malt broth (3, 3, 5, and 5 g/L of yeast extract, malt extract, peptone, and glucose, respectively [pH 6.2]) for 4 days at 30°C on an orbital shaker (100 rpm). Chromosomal DNA was isolated from a fungal pellet using a ZymoBIOMICS DNA miniprep kit (Zymo Research, USA), according to the manufacturer’s instructions, with an extended time of 40 min for continuous beating with beads (0.1 and 0.5 mm) on a Vortex 3 shaker (IKA, Staufen, Germany). DNA recovered in 100 μL of elution buffer was subjected to PCR amplification with the F1422/ITS7 primer set. PCR products purified using a High Pure PCR product purification kit (Roche Diagnostics International AG, Rotkreuz, Switzerland) were sequenced with the universal primers F1422, ITS1F, and ITS7. The obtained sequences were edited using Chromas Lite software (Technelysium Pty. Ltd., South Brisbane, QLD, Australia) and assembled and analyzed using the Lasergene software pack (DNAStar, Inc., Madison, WI, USA). Following the comparison of the final sequence to entries in the International Society of Human and Animal Mycology ITS reference DNA barcoding database, it was deposited in the GenBank database (accession number ON955910).

### Growth conditions and microscopy.

**(i) Germination.** A. fumigatus conidia were harvested in phosphate-buffered saline containing 0.01% Tween 80 from a culture grown at 37°C on malt extract agar (17, 3, and 20 g/L of malt extract, mycological peptone, and agar, respectively, adjusted to pH 5.4 before sterilization), and the suspension was filtered using a 5-μm SyringeStrainer (Pluriselect, San Diego, CA, USA). Next, A. fumigatus conidia (10^8^ per mL) were inoculated into iron-limited minimal medium (14.62, 3, 0.5, and 1 g/L of Na_2_HPO_4_·12H_2_O, KH_2_PO_4_, NaCl, and NH_4_Cl, respectively [pH 7]) supplemented with 5 g/L glucose as a source of carbon and trace elements (200, 50, 10, 17, 4.8, 3, and 4.5 mg/L of MgSO_4_·7H_2_O, CaCl_2_·2H_2_O, ZnSO_4_·7H_2_O, MnSO_4_·H_2_O, CoCl_2_·6H_2_O, CuSO_4_·5H_2_O, and Na_2_MoO_4_, respectively), and 10-mL cultures were shaken in 50-mL Erlenmeyer flasks on an orbital shaker (190 rpm) at 37°C for 72 h. The experiment was performed with four biological replicates. To detect fungal metabolites, samples of conidia, the residual fungal mass, and the supernatant were collected from each biological replicate at incubation times of 0, 3, 5, 6, 7, 8, 9, 10, 12, 14, 18, 24, 48, and 72 h. Before further sample processing, the supernatants were filtered through a Whatman membrane (5 μm; VWR International, Stříbrná Skalice, Czechia) using a sterile syringe. The residual fungal mass was washed three times with sterile water, centrifuged (14,000 × *g* for 2 min) at room temperature, and lyophilized.

**(ii) Microscopy.** Samples of 60 to 100 conidia collected up to 10 h of incubation were fixed in 37% formaldehyde. Their germination was observed using a DN45 light microscope (Lambda Praha Ltd., Prague, Czechia), and images were captured with an Eos 700D digital single-lens reflex (SLR) camera (Canon, Inc., Tokyo, Japan). The recorded images were calibrated using the Fiji software suite (42) with a stage objective micromanipulator.

### Extraction and quantitation of metabolites.

To extract siderophores (Fc, Hfc, FsC, TafC, and TafB) and mycotoxins (Gtx, fumigaclavine A, fumiquinazoline C, fumiquinazoline D, 3-hydroxy-fumiquinazoline A, and tryptoquivaline F/J) from A. fumigatus, a previously published protocol was used (29). Briefly, samples (50 μL of conidia, the residual fungal mass suspension, or the supernatant), all spiked with a FoxE (50 ng/mL) internal standard and ferrated with FeCl_3_ (100 μM), were subjected to two-step liquid-liquid extraction using ethyl acetate (150 μL) and MeOH (200 μL). Calibration standards of Fc, TafC, Gtx, fumigaclavine A, and fumiquinazoline D were prepared at final concentrations of 0.5, 1, 5, 10, 50, 100, 500, 750, and 1,000 ng/mL.

Siderophores and mycotoxins were extracted from human urine and serum samples according to a previously reported protocol for metabolic profiling (43). Briefly, urine and serum samples from patients and healthy individuals stored at −80°C were allowed to thaw at room temperature and then centrifuged at 6,000 rpm for 30 s. All centrifuged samples (50 μL) were spiked with the FoxE (100 ng/mL) internal standard. Urine and serum samples from healthy individuals were used to prepare calibration standards of Fc, TafC, and Gtx at final concentrations of 0.1, 0.5, 1, 5, 10, 50, 100, 500, 1,000, and 5,000 ng/mL. The prepared samples were loaded onto Sep-Pac C_18_ 1-mL Vac solid-phase extraction (SPE) cartridges (Waters, Prague, Czechia) that had been preconditioned with MeOH–0.1% FA and equilibrated with H_2_O–0.1% FA. Polar impurities were removed with 200 μL of 2% MeOH–0.1% FA, and the compounds of interest were eluted with 400 μL of MeOH–0.1% FA. The extracts from both the *in vitro* and human samples were vacuum dried for 2 h at 35°C using a SpeedVac (catalog number SPD121P; Thermo Scientific, Pardubice, Czechia) and stored at −80°C until HPLC-MS analysis.

### HPLC-MS analysis.

Before the HPLC-MS analysis in triplicates, all *in vitro* and human samples were reconstituted in 150 μL of 15% and 5% ACN, respectively. Siderophores and mycotoxins were separated using a Dionex UltiMate 3000 HPLC system (Thermo Scientific, MA, USA). Reconstituted urine and serum samples were injected into an Acquity high-strength silica (HSS) C_18_/1.8-μm, 2.1 by 5-mm VanGuard precolumn for precleaning and preconcentration, which was connected to an Acquity HSS T3/1.8-μm, 1.0 by 150-mm analytical column (both from Waters, Prague, Czechia). Analytes were eluted at a 50-μL/min flow rate using the following gradient of buffers A and B: 1% B for 1 min followed by linear increases to 60% B at 20 min and 99% B at 23 min and then a 3-min hold at 99% B, with a 2-min linear fall to 1% B and a 12-min hold to reequilibrate the column before the next injection. Here, buffer A was 5% ACN with 0.1% aqueous FA, and buffer B was 95% ACN with 0.1% aqueous FA. Previously reported LC-MS settings were applied for the analysis of samples from the *in vitro* experiments (29).

Siderophores and mycotoxins were detected using a SolariX 12T Fourier transform ion cyclotron resonance mass spectrometer (Bruker Daltonics, Billerica, MA, USA). Mass spectrometry data were collected in positive-ion mode with electrospray ionization, and MS parameters were adjusted to optimize the signal intensity of the analytes of interest by applying a quadrupole filter to facilitate the continuous accumulation of the selected ions at 100 to 700 and 500 to 1,500 *m/z* intervals for analyses of mycotoxins and siderophores, respectively.

### Data processing and method validation.

All acquired LC-MS data were processed using DataAnalysis v.5.0 software (Bruker Daltonics, Germany). Fumiquinazoline C, 3-hydroxy-fumiquinazoline A, and tryptoquivaline F/J were annotated from mass spectra by matching the respective exact *m/z* values and product ion mass spectra (see Fig. S2 in the supplemental material). The detected analytes were quantified using external calibration standards (see “Extraction and quantitation of metabolites,” above). Using extracted ion chromatograms with a 0.005-Da spectral width, the responses were summed from the integrated areas of the desferri- and ferri-forms of protonated, sodiated, and potassiated ion species (Table S4). The sum for each analyte was normalized to the integrated peak area of the internal standard FoxE. Assuming similar ionization efficiencies, Hfc was semiquantified using the Fc calibration curve, and fumiquinazoline C, 3-hydroxy-fumiquinazoline A, and tryptoquivaline F/J were semiquantified using the fumiquinazoline D calibration curve, while FsC and TafB were semiquantified using the TafC calibration curve. The results were averaged from triplicates. In the clinical study, the urine concentrations of siderophores (Fc, TafC, and TafB) and Gtx were further normalized to the urine creatinine concentration, to obtain creatinine index values (21), using the following formula: siderophore or secondary metabolite concentration (ng/mL)/creatinine concentration (mg/dL × 100).

Both *in vitro* and clinical sample preparation methods were validated using either control growth medium samples or control human urine samples, according to U.S. Food and Drug Administration guidelines for validating bioanalytical methods (44), in terms of the calibration curve (linearity), LOD, LOQ, intra- and interday accuracy and precision, selectivity, specificity, sensitivity, carryover, and autosampler stability. Instrumental LOD and LOQ values were defined as the lowest concentrations for which the SDs of the intercept equaled 3.3 and 10, respectively. The LOD, LOQ, linearity, and sensitivity were determined using a set of prepared nonzero calibration standards. The selectivity and specificity were determined using six randomly selected control human samples. The low- and high-concentration samples were separately prepared to determine the extraction efficiency (recovery), intra- and interday accuracy, and precision. The instrument’s performance was checked by a system suitability test using an HPLC peptide standard mixture (Sigma-Aldrich, Prague, Czechia). After analyzing the highest-concentration calibration standard, the carryover effect was evaluated for each analyte by injecting a blank sample. Independently prepared quality control samples were run throughout the studies and used to determine the reproducibility of the retention times and autosampler stability. Method validation parameters (LOD, LOQ, extraction recovery, carryover, sensitivity, and reproducibility) are summarized in Table S5. Inter- and intraday accuracy and precision and autosampler stability are specified in Table S6. Siderophore variations in biological replicates in the *in vitro* study are reported in Tables S7 to S9.

### Invasive pulmonary aspergillosis clinical study design.

The clinical study involved 35 patients aged 9 to 77 years, including 23 men and 12 women, admitted to intensive care units (ICUs) and respiratory departments at the University Hospital Ostrava and relevant departments of public health institutes in Ostrava between January 2018 and June 2020. For the diagnosis of IPA, the consensus definitions of invasive fungal diseases established by the EORTC/MSGERC ICU Working Group and guidelines of the European Society for Clinical Microbiology and Infectious Diseases and the European Committee for Medical Mycology for chronic pulmonary aspergillosis (CPA) were used (26, 45). Patients were divided into an IPA patient cohort of 13 patients and a control cohort of 22 patients (Fig. 3), with characteristics that included clinical findings, risk factors, and underlying diseases (Table 1; Tables S1 and S2). All 13 IPA cases were classified as probable aspergillosis with A. fumigatus found in the lower airways. No transbronchial biopsy specimen from a primarily sterile site of any patient was received. Urine and serum were sampled on the day of IPA diagnosis. This sampling was usually triggered by positive findings (serology or microscopy) obtained from the patients’ BALF or sputum samples, before the initiation of antifungal therapy. Four out of 13 patients died irrespective of the antimycotic treatment. Six control patients died from bacterial, yeast, or invasive fungal disease other than IPA. Aspergillus infection was not detected in any necropsy specimens in the control cohort. Together with clinical and mycological examinations of BALF cultures, patient serum samples were analyzed for BDG and GM at the site of the patient’s hospitalization. BDG measurements were performed using the Fungitell assay (assay range of <0.07 to 2,197 pg/mL; Associates of Cape Cod, Inc., USA). GM antigen detection was performed using the Aspergillus enzyme immunoassay (EIA) (Platelia; Bio-Rad, France). The use of urine and serum samples in the observational, noninterventional study was approved by the Ethics Committee for Clinical Trials of the Institute of Public Health (EC-02/18) and the University Hospital Ostrava (448/2018), and informed-consent documents were obtained from study participants. Analyses of patient urine and serum samples were performed using the LC-MS-based infection metallomics approach (24). Throughout the study, we strove to adhere to the good clinical practice guidelines outlined by the Declaration of Helsinki (2013). For all procedures involving the handling of potentially infectious material, the care of research staff conformed to general guidelines for protecting the European Community (46, 47).

### Statistical analysis.

The variations in the measured siderophores and mycotoxins from four biological replicates of *in vitro* A. fumigatus cultures at each sampling point during their germination and subsequent growth were characterized in terms of means, SDs, standard errors of the means, and coefficients of variation using MS Excel 2016 and graphically visualized using OriginPro version 22 software (OriginLab Corporation, Northampton, MA, USA). Data are presented as box plots displaying means ± standard deviations.

Data obtained from the analysis of clinical samples were statistically analyzed using GraphPad Prism 8.0.1 software (GraphPad, San Diego, CA, USA). The reported descriptive statistics include means, medians, interquartile ranges, SDs, standard errors of the means, and coefficients of variation (Table S10). The Gaussian distribution of the data was tested using the D’Agostino-Pearson normality test. Since the normality test did not meet the requirements for parametric tests, nonparametric tests were used. The statistical dependence between the rankings of individual siderophores and serological markers was assessed by Spearman correlation coefficients. For all of the above-mentioned tests, a *P* value of <0.05 was considered statistically significant. Test positivity cutoffs for urine fungal secondary metabolites and serological markers were defined using a receiver operating characteristic curve (Fig. S3). The urine and serum samples were considered positive if at least one of the urine creatinine-indexed fungal metabolite or serum concentrations (TafC, TafB, Fc, or Gtx) was higher than an LC-MS method-defined LOD for a particular marker. Using conventional clinical diagnostic approaches, serum samples were considered positive if the BDG concentrations or GM indices exceeded a cutoff value of 80 pg/mL or an optical density index (ODI) of 0.5, respectively. Sensitivity and specificity were calculated using the Clopper-Pearson confidence interval.

### Data availability.

The final sequence was deposited in the GenBank database under accession number ON955910. Raw data will be provided by the corresponding author upon reasonable request.
