# Supplementary material for: Siderophore-Based Noninvasive Differentiation of Aspergillus fumigatus Colonization and Invasion in Pulmonary Aspergillosis
Source: Microbiol Spectr. 2023 Jan 31;11(2):e04068-22. doi: 10.1128/spectrum.04068-22 (PMC10100950; doi:10.1128/spectrum.04068-22)
Supplement: Supplemental file 1 — Supplemental material. Download spectrum.04068-22-s0001.pdf, PDF file, 1.0 MB [file spectrum.04068-22-s0001.pdf]

SUPPLEMENTARY MATERIALS

Siderophore-based noninvasive differentiation of *Aspergillus fumigatus* colonization and invasion in pulmonary aspergillosis

Dominika Luptáková[1]†, Rutuja H. Patil[1,2]†, Radim Dobiáš[3,4], David A. Stevens[5,6], Tomáš Pluháček[1,2], Andrea Palyzová[1], Marcela Káňová[7-9], Milan Navrátil[10], Zbyněk Vrba[11], Petr Hubáček[12], Vladimír Havlíček[1*]

[1]Institute of Microbiology of the Czech Academy of Sciences; 142 20 Prague, Czechia.

[2]Department of Analytical Chemistry, Palacký University; 771 46 Olomouc, Czechia.

[3]Department of Bacteriology and Mycology, Public Health Institute in Ostrava; 702 00 Ostrava, Czechia.

[4]Institute of Laboratory Medicine, Faculty of Medicine, University of Ostrava; 703 00 Ostrava, Czechia.

[5]California Institute for Medical Research, San Jose, CA 95128, USA.

[6]Division of Infectious Diseases and Geographic Medicine, Stanford University School of Medicine; Stanford, USA.

[7]Department of Anesthesiology and Intensive Care Medicine, University Hospital Ostrava; 708 00 Ostrava, Czechia.

[8]Institute of Physiology and Pathophysiology, Faculty of Medicine, University of Ostrava; 710 00 Ostrava, Czechia.

[9]Department of Intensive Medicine, Emergency Medicine and Forensic Studies, University of Ostrava; 710 00 Ostrava, Czechia.

[10]Department of Hematooncology, University Hospital of Ostrava; 708 00 Ostrava, Czechia.

[11]Lung Department, Krnov Combined Medical Facility; 794 01 Krnov, Czechia.

[12]Department of Medical Microbiology, Charles University and Motol University Hospital; 150 06 Prague, Czechia.

*Corresponding author. Email: vlhavlic@biomed.cas.cz

† Equal contribution

Table of Contents

Fig. S1. Variations in *A. fumigatus* intracellular mycotoxin contents.

Fig. S2. Results of analyses of urine of immunocompromised and immunocompetent patients for the noninvasive detection of IPA.

Fig. S3. Identification and product ion mass spectra of mycotoxins.

Fig. S4. Receiver operating characteristics (ROC) for *Aspergillus* biomarkers performance challenged in urine and serum.

Table S1. Characteristics of patients in the control (non-IPA) group.

Table S2. *Aspergillus* biomarker performance in the human serum.

Table S3. Spearman's correlation coefficients (r) among urine and serum biomarkers detected in the patient cohort diagnosed with probable invasive pulmonary aspergillosis.

Table S4. Calculated isotopic masses of siderophore ions observed in clinical and *in vitro* data.

Table S5. Method validation parameters.

Table S6. Inter- and intra-day accuracy and precision, and autosampler stability.

Table S7. Variations in biomarker concentrations in conidia probed by the coefficients of variation.

Table S8. Variations in biomarker concentrations in supernatants challenged with the coefficients of variation.

Table S9. Variations in biomarker concentrations in *Aspergillus* pellets probed by coefficients of variation.

Table S10. Variations in *Aspergillus* biomarkers detected in urine and sera of IPA-patients probed with the coefficients of variation.

Supplemental Movie. Af germination.mp4. 16MB supplementary videofile indicating the time course of *A. fumigatus* conidial germination.

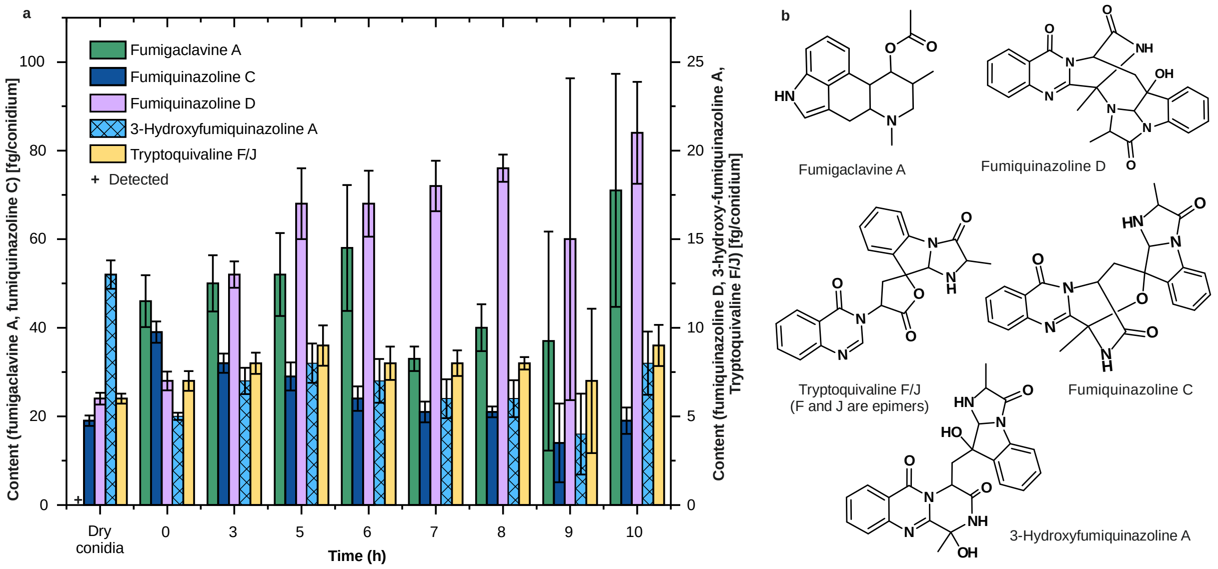

Fig. S1. Variations in *A. fumigatus* intracellular mycotoxin contents. Results of quantitative and semiquantitative analysis of five detected toxins in conidia (**a**) and their molecular structures (**b**). Bar graphs represent the averages of four biological replicates. Data are presented as mean ± standard deviation. J and Tryptoquivaline F are epimers (Yamazaki, M., Fujimoto, H. & Okuyama, E. Structure determination of 6 fungal metabolites, Tryptoquivaline E, F, G, H, I and J from *Aspergillus fumigatus*. *Chem Pharm Bull* **26**, 111-117 (1978)).

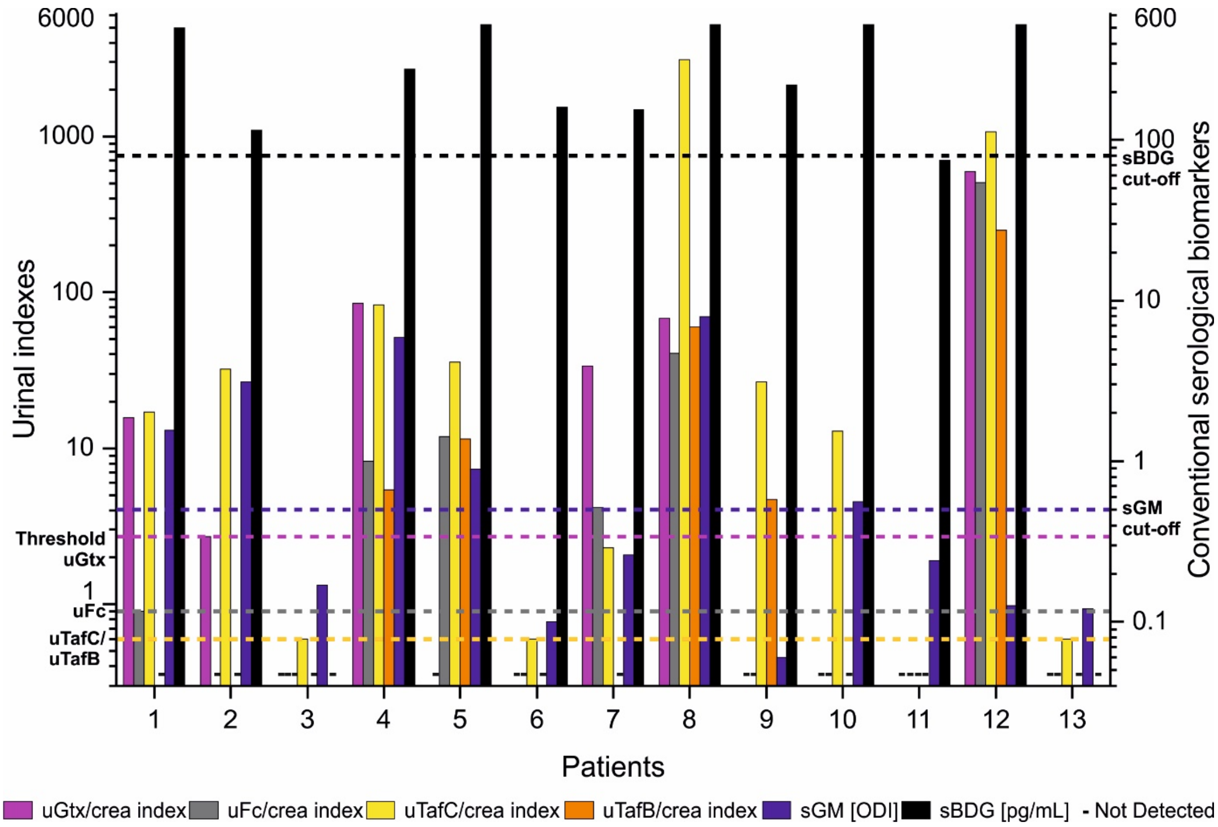

Fig. S2. Results of analyses of urine of immunocompromised and immunocompetent patients for the noninvasive detection of IPA. All cases (13 in total) were classified as “probable aspergillosis” patients (references 24, 25 in the main manuscript). u, urine; s, serum; Fc, ferricrocin; TafC, triacetylfusarinine C; TafB, triacetylfusarinine B; Gtx, gliotoxin; GM, galactomannan; BDG, β-D-glucan; -, not detected; urine index definition (reference 21 in the main manuscript).

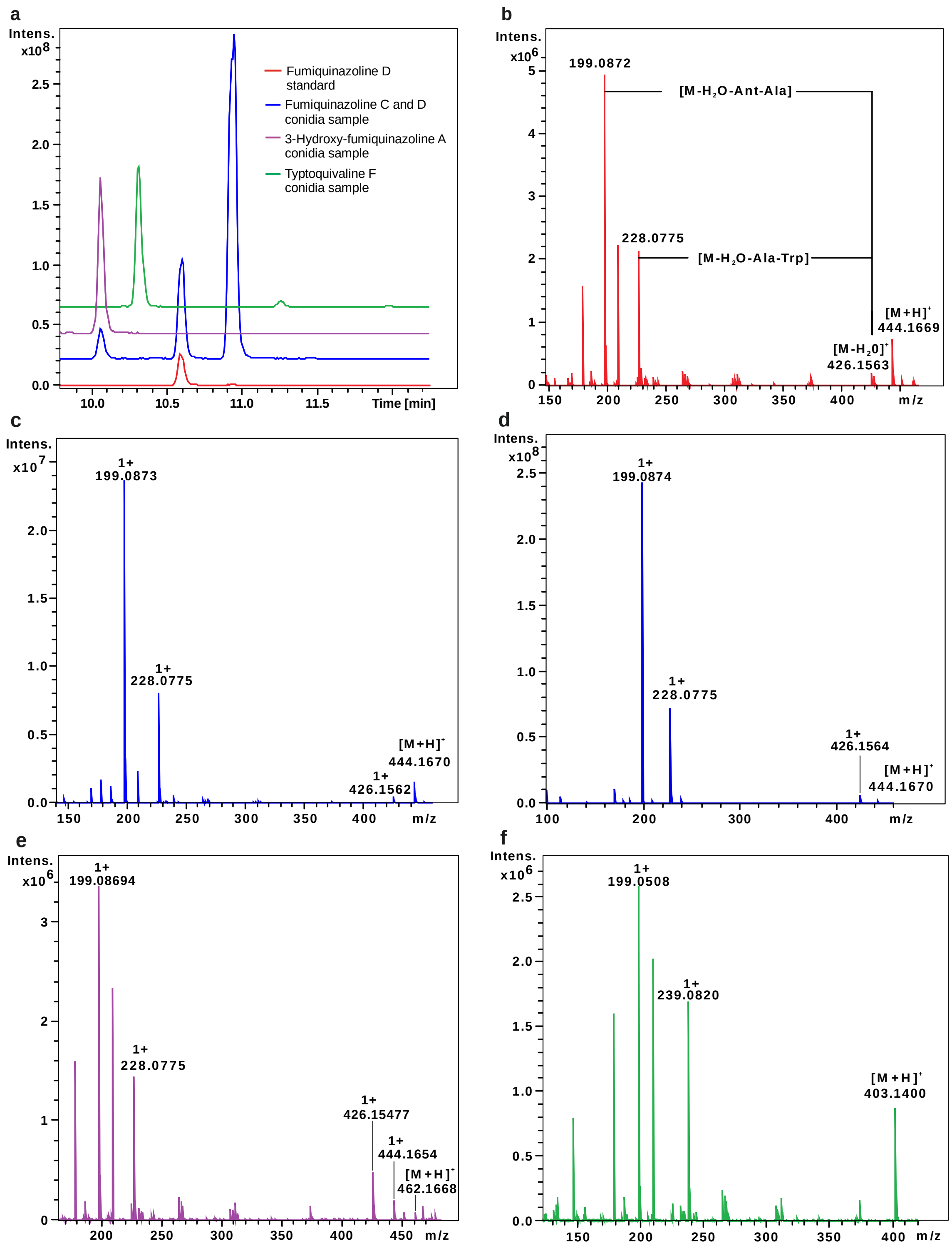

Fig. S3. Identification and product ion mass spectra of mycotoxins. (a) Extracted-ion chromatograms of mycotoxins from standard and conidial samples. **(b-f)** Fragmentation mass spectra of: **(b)** fumiquinazoline D from a standard, **(c)** fumiquinazoline D from a conidial sample, **(d)** fumiquinazoline C from a conidial sample, **(e)** 3-hydroxy-fumiquinazoline A from a conidial sample, and **(f)** tryptoquivaline F/J from a conidial sample. Ant, anthranilic acid; Ala, alanine; Trp, tryptophan. Tryptoquivaline J and Tryptoqualine F are epimers (Yamazaki, M., Fujimoto, H. & Okuyama, E. Structure determination of 6 fungal metabolites, Tryptoquivaline E, F, G, H, I and J from *Aspergillus fumigatus*. *Chem Pharm Bull* **26**, 111-117 (1978)).

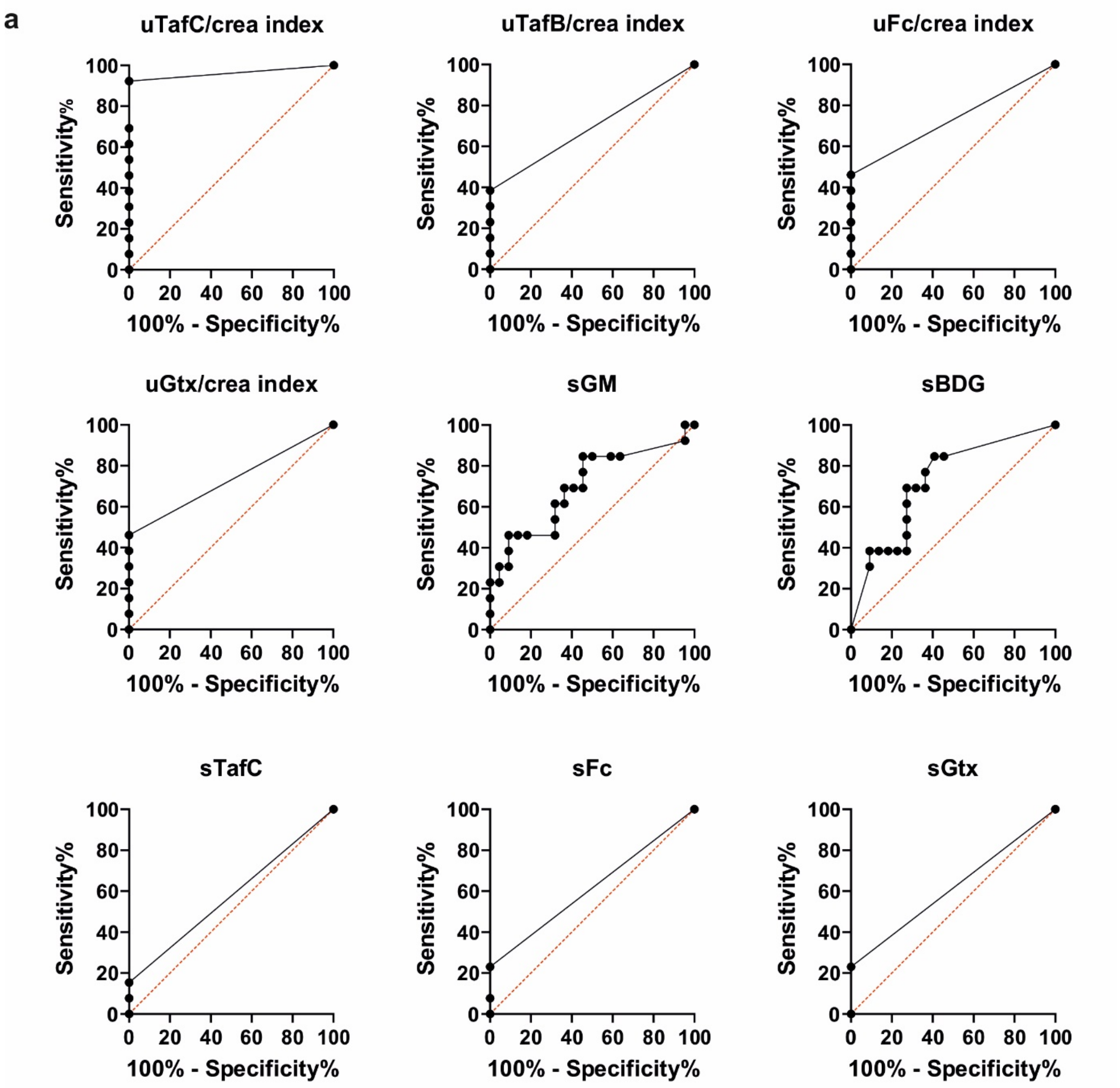

b

Area under the ROC curve	uGtx/crea index*	uFc/crea index*	uTafC/crea index*	uTafB/crea index*	sGtx	sFc	sTafC	sGM	sBDG
**Area**	0.7308	0.7308	0.9615	0.6923	0.6154	0.6154	0.5769	0.7010	0.7255
**SE**	0.0978	0.0978	0.0439	0.1009	0.1042	0.1042	0.1045	0.0966	0.0892
**95% CI**	53.9 – 92.2	53.9 – 92.2	87.6 - 100	49.5 – 89	41.1 – 82	41.1 – 82	37.2 – 78.2	51.2 – 89	55.1 – 90
***p*-value**	0.0242	0.0242	<0.0001	0.0604	0.2599	0.2599	0.4526	0.0496	0.0277

Fig. S4. Receiver operating characteristics (ROC) for *Aspergillus* biomarkers performance challenged in urine and serum. (a) The ROC curves representing the combination of sensitivity and specificity for each possible test positivity cut-off. **(b)** Descriptive statistics of area under the ROC curve, including standard error (SE), 95% confidence interval (CI), and statistical significance expressed as a *p*-value**.** *, creatinine index, previously defined (Hoenigl, M. et al. Triacetylfusarinine C: A urine biomarker for diagnosis of invasive aspergillosis. J Infect 78, 150-157 (2019)); crea, creatinine; u, urine; s, serum; Fc, ferricrocin; TafC, triacetylfusarinine C; TafB, triacetylfusarinine B; Gtx, gliotoxin; GM, galactomannan; BDG, β-D-glucan.

Table S1. Characteristics of patients in the control (non-IPA) group.
*, creatinine index, previously defined (Hoenigl, M. *et al*. Triacetylfusarinine C: A urine biomarker for diagnosis of invasive aspergillosis. *J Infect* **78**, 150-157 (2019)); u, urine; s, serum; Fc, ferricrocin; TafC, triacetylfusarinine C; TafB, triacetylfusarinine B; Gtx, gliotoxin; GM, galactomannan; BDG, β-D-glucan; ODI, optical density index; COPD, Chronic Obstructive Pulmonary disease; GIT, Gastrointestinal Tract; AML, Acute Myeloblastic Leukemia; TPL, Transplantation; AFcol, *Aspergillus fumigatus* lower respiratory tract colonization; CPA, Chronic Pulmonary Aspergillosis; ABPA, Allergic Broncho-Pulmonary Aspergillosis; AFLA, *Aspergillus flavus*; ANI, *Aspergillus niger*; CAcol, *Candida albicans* colonization; CDUcol, *Candida dubliniensis* colonization; IC, Invasive Candidiasis; CGL, *Candida glabrata*; CTR, *Candida tropicalis*; MM, Mucormycosis; RMICR, *Rhizopus microsporus*; Rcol, *Rhizopus* sp. colonization; LCORcol, *Lichtheimia corymbifera* colonization; CM, Cutaneous Mucormycosis; MCIRC, *Mucor circinelloides*; SAcol, *Scedosporium apiospermum* colonization, RMICRcol, *Rhizopus microsporus* colonization; PSAE, *Pseudomonas aeruginosa*; PSAEcol, *Pseudomonas aeruginosa* colonization; M, male; F, female; nd, not detected; NA, information not available.

**Underlying disease**	**Clinical finding**	**Sex**	**Age**	***Infection Metallomics***				**Conventional approaches**		**Death**
				**uGtx/crea index***	**uFc/crea index***	**uTafC/crea index***	**uTafB/crea index***	**sGM (ODI)**	**sBDG (pg/mL)**	
COPD III.	AFcol	M	76	nd	nd	nd	nd	0.37	128	No
Asthma	CPA – AF	F	70	nd	nd	nd	nd	0.05	nd	No
NA	ABPA – AFLA	F	63	nd	nd	nd	nd	0.129	nd	No
Sarcoidosis	CPA – AFLA	F	49	nd	nd	nd	nd	0.5	75	No
COPD	CPA – ANI	M	44	nd	nd	nd	nd	1.85	523	No
Bronchpneumonia	CAcol	F	74	nd	nd	nd	nd	NA	55	No
Polytrauma	CDUcol	M	42	nd	nd	nd	nd	NA	143	No
Peritonitis	IC – CDU	F	49	nd	nd	nd	nd	0.104	365	Yes
Surgery/upper GIT	IC – CGL	F	22	nd	nd	nd	nd	1.48	322	Yes
Peritonitis	IC – CGL	F	53	nd	nd	nd	nd	NA	344	Yes
Sepsis	IC – CTR	M	76	nd	nd	nd	nd	NA	523	No
AML	MM-RMICR	F	38	nd	nd	nd	nd	0.12	nd	No
TPL - kidney	MM-RMICR	M	60	nd	nd	nd	nd	0.16	nd	Yes
Polytrauma	Rcol	M	57	nd	nd	nd	nd	0.2	nd	No
COPD	LCORcol	F	57	nd	nd	nd	nd	0.3	nd	No
Polytrauma	CM-MCIRC	M	77	nd	nd	nd	nd	0.11	nd	No
Polytrauma	SAcol	F	29	nd	nd	nd	nd	0.11	440	No
NA	Rcol	M	9	nd	nd	nd	nd	0.3	nd	NA
NA	RMICRcol	M	NA	nd	nd	nd	nd	0.3	nd	NA
Polytrauma	PSAE sepsis	M	42	nd	nd	nd	nd	NA	nd	Yes
TPL - kidney	PSAE sepsis	M	60	nd	nd	nd	nd	NA	nd	Yes
NA	PSAEcol	M	43	nd	nd	nd	nd	NA	NA	NA

Table S2: *Aspergillus* biomarker performance in the human serum. Strikingly lower performance in sensitivity recorded for triacetylfusarinine C (TafC), ferricrocin (Fc), and mycotoxin gliotoxin (Gtx) in the IPA patient's sera contrasting with the premium performance in the respective urine samples. s, serum; COPD, Chronic Obstructive Pulmonary disease; DM II, Diabetes Mellitus II; IST, immunosuppressants; DC, decompensated cirrhosis; CI, confidence interval; Flu, influenza, M, male; F, female; nd, not detected; det, detected. Threshold represents the limit of detection of the LC/MS method for the level of serum biomarkers expressed in ng/mL.

**Underlying disease**	**Risk factor**	**Sex**	**Age**	***Infection Metallomics***		
				**sGtx**	**sFc**	**sTafC**
Multiple myeloma	Neutropenia, IST	M	50	nd	nd	nd
Non-Hodgkin lymphoma	Neutropenia, IST	F	66	nd	nd	nd
Multiple myeloma	Neutropenia, IST	M	56	nd	nd	nd
Bronchopneumonia	Flu (H1N1)	M	79	det	29.1±1.7	18.0±0.9
COPD	Steroids	M	71	nd	nd	nd
COPD	Flu (H1N1)	F	66	nd	nd	nd
DM II	Ketoacidosis	M	50	nd	nd	nd
Bronchopneumonia	Flu (H1N1), Steroids	M	65	det	det	114.7±2.0
Liver transplant	Steroids, IST	M	59	nd	nd	nd
Bronchopneumonia	Steroids	M	61	nd	nd	nd
Polytrauma		M	51	nd	nd	nd
COPD	DC	M	68	det	det	nd
Burns		M	75	nd	nd	nd
Threshold (ng/mL)				≥4.5	≥1.8	≥0.7
Sensitivity % (95%CI)				23.1 (5.0 – 53.8)	23.1 (5.0 – 53.8)	15.4 (1.9 – 45.4)
Specificity % (95%CI)				100 (84.6 – 100)	100 (84.6 – 100)	100 (84.6 – 100)

Table S3. Spearman's correlation coefficients (*r*) among urine and serum biomarkers detected in the patient cohort diagnosed with probable invasive pulmonary aspergillosis. The results were considered statistically significant if $p \leqslant 0.05$. *, creatinine index, as previously defined (Hoenigl, M. *et al.* Triacetylfusarinine C: A urine biomarker for diagnosis of invasive aspergillosis. *J Infect* **78**, 150-157 (2019)); ODI, optical density index; u, urine; s, serum; Fc, ferricrocin; TafC, triacetylfusarinine C; TafB, triacetylfusarinine B; Gtx, gliotoxin; GM, galactomannan; BDG, β-D-glucan.

r	**uGtx/crea index***	**uFc/crea index***	**uTafC/crea index***	**uTafB/crea index***	**sGM (ODI)**	**sBDG (pg/mL)**
**uGtx/crea index***	-					
**uFc/crea index***	0.766	-				
**uTafC/crea index***	0.691	0.769	-			
**uTafB/crea index***	0.514	0.800	0.841	-		
**sGM (ODI)**	0.514	0.460	0.541	0.207	-	
**sBDG (pg/mL)**	0.400	0.679	0.743	0.679	0.379	-

p	**uGtx/crea index***	**uFc/crea index***	**uTafC/crea index***	**uTafB/crea index***	**sGM (ODI)**	**sBDG (pg/mL)**
**uGtx/crea index***	-					
**uFc/crea index***	0.0031	-				
**uTafC/crea index***	0.0118	0.0037	-			
**uTafB/crea index***	0.0690	0.0020	0.0005	-		
**sGM (ODI)**	0.0762	0.1165	0.0595	0.4945	-	
**sBDG (pg/mL)**	0.1738	0.0138	0.0053	0.0130	0.1998	-

Table S4. Calculated isotopic masses of siderophore ions observed in clinical and *in vitro* data. Fc, ferricrocin; Hfc, hydroxyferricrocin; TafC, triacetylfusarinine C; TafB, triacetylfusarinine B; FsC, fusarinine C; FoxE, ferrioxamine E; Gtx, gliotoxin; M, molecule; +H, proton adduct; +Na, sodium adduct; +K, potassium adduct; +Fe, ferri-form of the molecule. Tryptoquivaline J and Tryptoqualine F are epimers (Yamazaki, M., Fujimoto, H. & Okuyama, E. Structure determination of 6 fungal metabolites, Tryptoquivaline E, F, G, H, I and J from *Aspergillus fumigatus*. *Chem Pharm Bull* **26**, 111-117 (1978)).

Analytes	Molecular formula	$[M+H]^+$	$[M+Na]^+$	$[M+K]^+$	$[M+Fe-2H]^+$	$[M+Fe+Na-3H]^+$	$[M+Fe+K-3H]^+$
**Fc**	$C_{28}H_{47}N_9O_{13}$	718.3366	740.3186	756.2925	771.2481	793.2301	809.2040
**Hfc**	$C_{28}H_{47}N_9O_{14}$				787.2430	809.2249	825.1989
**TafC**	$C_{39}H_{60}N_6O_{15}$	853.4189	875.4009	891.3748	906.3305	928.3124	944.2864
**TafB**	$C_{39}H_{62}N_6O_{16}$	871.4295	893.4115	909.3854	924.3411	946.3230	962.2970
**FsC**	$C_{33}H_{54}N_6O_{12}$	727.3872	749.3692	765.3431	780.2988	802.2807	818.2547
**FoxE**	$C_{27}H_{48}N_6O_9$				654.2671		
**Gtx**	$C_{13}H_{14}N_2O_4S_2$	327.0468	349.0287	365.0027			
**Fumiquinazoline D**	$C_{24}H_{21}N_5O_4$	444.1666	466.1486	482.1225			
**Fumigaclavine A**	$C_{18}H_{22}N_2O_2$	299.1754	321.1573	337.1313			
**Fumiquinazoline C**	$C_{24}H_{21}N_5O_4$	444.1666	466.1486	482.1225			
**3-Hydroxy-fumiquinazoline A**	$C_{24}H_{23}N_5O_5$	462.1853	484.1591	500.1331			
**Tryptoquivaline F/J**	$C_{22}H_{18}N_4O_4$	403.1401	425.1220	441.0960			

Table S5. Method validation parameters. (a) clinical urine samples and (b) *in vitro* samples, including method limit of detection and quantitation, extraction recovery, carryover, sensitivity and reproducibility. #, number of sample blanks injected after the highest concentrated calibration standard = 4; Fc, ferricrocin; TafC, triacetylfusarinine C; Gtx, gliotoxin; LOD, limit of detection; LOQ, limit of quantification; LL, low level concentration 10 ng/mL; HL, high level concentration 500 ng/mL; * low level concentration 500 ng/mL, high level concentration 2500 ng/mL; RSD, relative standard deviation.

a

Parameters	Analytes		
	Fc	TafC	Gtx*
**LOD (ng/mL)**	0.9	0.6	2.7
**LOQ (ng/mL)**	3.0	1.5	8.4
**LL extraction recovery (%)**	78	94	90
**HL extraction recovery (%)**	97	107	102
**Carryover (%)#**	0.2 ± 0.1	0.2 ± 0.1	0.04 ± 0.06
**Reproducibility of retention time (RSD, %)**	0.6	0.3	0.2

b

Parameters	Analytes				
	Fc	TafC	Gtx	Fumiquinazoline D	Fumigaclavine A
**LOD (ng/mL)**	1.9	1.8	0.8	3.8	12.2
**LOQ (ng/mL)**	5.8	5.4	2.2	11.5	37.1
**LOD (fg/conidium)**	0.5	0.4	0.2	0.9	3.1
**LOQ (fg/conidium)**	1.4	1.3	0.6	2.9	9.3
**LOD (µg/g)**	0.4	0.3	0.1	0.7	2.3
**LOQ (µg/g)**	1.1	1.0	0.4	2.2	7.0
**Reproducibility of retention time (RSD, %)**	<0.01	<0.01	0.05	<0.01	0.04

Table S6. Inter- and intra-day accuracy and precision, and autosampler stability. Fc, ferricrocin; TafC, triacetylfusarinine C; Gtx, gliotoxin; LL, low level concentration 10 ng/mL; HL, high level concentration 500 ng/mL; * low level concentration 500 ng/mL, high level concentration 2500 ng/mL; RSD, relative standard deviation.

Study	Analyte	Level	Concentration (ng/mL)	Day 1 Accuracy (%)	Day 1 Precision (%)	Day 2 Accuracy (%)	Day 2 Precision (%)	Day 3 Accuracy (%)	Day 3 Precision (%)	Mean Inter-day Accuracy (%)	Mean Inter-day Precision (%)
Clinical	**FC**	LL	10	102	5	103	3	108	3	104	3
		HL	500	103	1	105	1	102	1	103	1
	**TafC**	LL	10	115	2	116	2	120	3	117	2
		HL	500	119	3	115	1	120	4	118	3
	**GtX**	LL	500	109	3	108	2	109	2	109	2
		HL	2 500	107	4	104	2	96	9	102	5
In vitro	**FC**	LL	25	97	2	105	1	115	2	106	2
		HL	400	109	1	113	2	120	5	114	3
	**TafC**	LL	25	99	5	114	4	96	4	103	4
		HL	400	147	5	153	4	142	7	147	5
	**GtX**	LL	100	105	11	116	14	81	2	101	10
		HL	400	80	1	81	3	101	8	88	4
	**bmGtX**	LL	100	92	4	115	10	89	4	99	6
		HL	400	71	2	71	4	84	6	75	4
	**Fumiquinazoline D**	LL	25	89	7	93	3	104	5	96	5
		HL	400	96	2	101	3	114	6	104	4
	**Fumigaclavine A**	LL	25	74	7	72	4	79	5	75	5
		HL	400	71	3	69	6	77	6	72	5

Table S7. Variations in biomarker concentrations in conidia probed by the coefficients of variation (CV). SEM, standard error of mean; SD, standard deviation; nd, not detected; det, detected; NaN, not a number (used for “nd” cases and mean concentrations between the limit of detection and limit of quantitation); Fc, ferricrocin; Hfc, hydroxyferricrocin; TafC, triacetylfusarinine C; TafB, triacetylfusarinine B.

Analytes	Conidia	0 h	3 h	5 h	6 h	7 h	8 h	9 h	10 h
**Fc**									
Mean (fg/conidium)	nd	nd	nd	1.95	5.31	8.27	29.44	110.50	492.31
SD (fg/conidium)	NaN	NaN	NaN	0.52	0.72	1.47	10.35	67.73	81.61
±SEM (fg/conidium)	NaN	NaN	NaN	0.15	0.21	0.42	2.99	19.55	23.56
CV	NaN	NaN	NaN	0.264	0.136	0.177	0.352	0.613	0.166
**Hfc**									
Mean (fg/conidium)	5.07	2.98	2.46	1.56	1.48	det	det	det	1.67
SD (fg/conidium)	0.03	0.62	0.17	0.21	0.07	NaN	NaN	NaN	0.30
±SEM (fg/conidium)	0.02	0.18	0.05	0.06	0.02	NaN	NaN	NaN	0.09
CV	0.006	0.207	0.070	0.136	0.050	NaN	NaN	NaN	0.180
**TafC**									
Mean (fg/conidium)	nd	nd	nd	nd	nd	nd	4.53	103.58	1289.18
SD (fg/conidium)	NaN	NaN	NaN	NaN	NaN	NaN	3.37	62.72	482.87
±SEM (fg/conidium)	NaN	NaN	NaN	NaN	NaN	NaN	0.97	18.11	139.39
CV	NaN	NaN	NaN	NaN	NaN	NaN	0.745	0.606	0.375
**TafB**									
Mean (fg/conidium)	nd	nd	nd	nd	nd	nd	nd	9.19	133.32
SD (fg/conidium)	NaN	NaN	NaN	NaN	NaN	NaN	NaN	8.14	24.68
±SEM (fg/conidium)	NaN	NaN	NaN	NaN	NaN	NaN	NaN	2.35	7.12
CV	NaN	NaN	NaN	NaN	NaN	NaN	NaN	0.885	0.185
**Fusarinine C**									
Mean (fg/conidium)	nd	nd	nd	nd	2.30	3.91	8.91	19.34	18.06
SD (fg/conidium)	NaN	NaN	NaN	NaN	1.64	0.47	3.49	4.79	6.87
±SEM (fg/conidium)	NaN	NaN	NaN	NaN	0.47	0.13	1.01	1.38	1.98
CV	NaN	NaN	NaN	NaN	0.712	0.119	0.391	0.247	0.380
**Fumigaclavine A**									
Mean (fg/conidium)	det	45.51	49.70	51.50	57.83	33.03	39.89	36.71	71.39
SD (fg/conidium)	NaN	5.85	6.35	9.35	14.21	2.76	5.28	24.71	26.32
±SEM (fg/conidium)	NaN	1.69	1.83	2.70	4.10	0.80	1.52	7.13	7.60
CV	NaN	0.129	0.128	0.182	0.246	0.084	0.132	0.673	0.369
**Fumiquinazoline D**									
Mean (fg/conidium)	6.24	6.95	12.96	16.64	16.92	17.76	18.56	14.74	20.61
SD (fg/conidium)	0.33	0.53	0.75	2.00	1.86	1.42	0.77	9.08	2.88
±SEM (fg/conidium)	0.19	0.15	0.22	0.58	0.54	0.41	0.22	2.62	0.83
CV	0.054	0.076	0.058	0.120	0.110	0.080	0.041	0.111	0.140
**Fumiquinazoline C**									
Mean (fg/conidium)	19.48	38.90	31.88	28.59	24.03	21.07	20.52	14.57	19.06
SD (fg/conidium)	1.18	2.41	2.18	3.17	2.76	2.34	1.22	8.49	2.98
±SEM (fg/conidium)	0.68	0.69	0.63	0.92	0.80	0.68	0.35	2.45	0.86
CV	0.061	0.062	0.068	0.111	0.115	0.111	0.059	0.583	0.156

**3-hydroxyfumiquinazoline A**									
Mean (fg/conidium)	12.97	5.26	6.73	8.14	7.43	6.10	6.36	3.98	7.80
SD (fg/conidium)	0.80	0.20	0.74	1.11	1.23	1.10	1.04	1.86	1.78
$\pm SEM$ (fg/conidium)	0.461	0.059	0.214	0.319	0.356	0.316	0.301	0.536	0.515
CV	0.062	0.032	0.110	0.136	0.166	0.180	0.164	0.467	0.228
**Tryptoquivaline F/J**									
Mean (fg/conidium)	5.58	7.41	7.71	8.66	8.49	8.10	8.37	6.80	9.23
SD (fg/conidium)	0.28	0.56	0.60	1.14	0.94	0.72	0.35	3.66	1.16
$\pm SEM$ (fg/conidium)	0.162	0.161	0.173	0.328	0.273	0.207	0.101	1.06	0.334
CV	0.050	0.075	0.078	0.131	0.111	0.089	0.042	0.538	0.125

Table S8. Variations in biomarker concentrations in supernatants challenged with the coefficients of variation (CV). SEM, standard error of mean; SD, standard deviation; nd, not detected; det, detected; NaN, not a number (used for “nd” cases and mean concentrations between the limit of detection and limit of quantitation); Fc, ferricrocin; TafC, triacetylfusarinine C; TafB, triacetylfusarinine B.

Analytes	12h	14h	18h	24h	48h	72h
**Fc**						
Mean (µg/mL)	3.08	4.04	9.94	16.43	21.31	31.51
SD (µg/mL)	0.27	0.28	1.48	1.73	3.83	3.62
±SEM (µg/mL)	0.08	0.08	0.43	0.50	1.10	1.04
CV	0.087	0.070	0.149	0.105	0.180	0.115
**TafC**						
Mean (µg/mL)	35.55	64.43	244.39	386.38	57.48	0.11
SD (µg/mL)	4.30	4.29	23.27	68.12	38.92	0.02
±SEM (µg/mL)	1.24	1.24	6.72	19.66	11.23	0.01
CV	0.121	0.067	0.095	0.176	0.677	0.204
**TafB**						
Mean (µg/mL)	0.21	0.54	2.51	35.20	214.64	11.77
SD (µg/mL)	0.03	0.12	0.31	18.90	57.69	9.13
±SEM (µg/mL)	0.01	0.03	0.09	5.456	16.655	2.634
CV	0.137	0.216	0.124	0.537	0.269	0.775

Table S9. Variations in biomarker concentrations in *Aspergillus* pellets probed by coefficients of variation (CV). SEM, standard error of mean; SD, standard deviation; nd, not detected; det, detected; NaN, not a number (used for "nd" cases and mean concentrations between the limit of detection and limit of quantitation); Fc, ferricrocin; Hfc, hydroxyferricrocin; TafC, triacetylfusarinine C; TafB, triacetylfusarinine B; FsC, fusarinine C.

Analytes	12 h	14 h	18 h	24 h	48 h	72 h
**Fc**						
Mean (µg/g)	2159	1821	2924	1618	2576	2944
SD (µg/g)	731	630	957	1038	1079	1050
±SEM (µg/g)	211	182	276	300	312	303
CV	0.339	0.346	0.327	0.642	0.419	0.357
**Hfc**						
Mean (µg/g)	38.03	nd	nd	nd	384	955
SD (µg/g)	10.72	NaN	NaN	NaN	149	255
±SEM (µg/g)	3.10	NaN	NaN	NaN	43	73
CV	0.282	NaN	NaN	NaN	0.389	0.267
**TafC**						
Mean (µg/g)	419	889	2099	3875	1231	nd
SD (µg/g)	29	211	1393	1263	631	NaN
±SEM (µg/g)	8	61	402	365	182	NaN
CV	0.070	0.238	0.664	0.326	0.513	NaN
**TafB**						
Mean (µg/g)	78.35	70.45	208	531	2159	283
SD (µg/g)	21.08	22.22	119	212	762	311
±SEM (µg/g)	6.08	6.42	34	61	220	90
CV	0.269	0.315	0.571	0.399	0.353	1.099
**FsC**						
Mean (µg/g)	5.42	7.38	det	nd	nd	nd
SD (µg/g)	1.91	4.32	NaN	NaN	NaN	NaN
±SEM (µg/g)	0.553	1.247	NaN	NaN	NaN	NaN
CV	0.353	0.586	NaN	NaN	NaN	NaN

Table S10. Variations in *Aspergillus* biomarkers detected in urine and sera of IPA-patients probed with the coefficients of variation (CV). *, creatinine index as previously published (Hoenigl, M. et al. Triacetylfusarinine C: A urine biomarker for diagnosis of invasive aspergillosis. J Infect 78, 150-157 (2019)); ODI, optical density index; u, urine; s, serum; Fc, ferricrocin; TafC, triacetylfusarinine C; TafB, triacetylfusarinine B; Gtx, gliotoxin; GM, galactomannan; BDG, β-D-glucan; SEM, standard error of mean; SD, standard deviation; IQR, interquartile range.

	**uTafC/crea index***	**uTafB/crea index***	**uFc/crea index***	**uGtx/crea index***	**sGtx**	**sFc**	**sTafC**	**sGM (ODI)**	**sBDG (pg/mL)**
Median	17.2	0.6	0.9	2.7	4.5	1.8	0.7	0.26	221.0
IQR	1.5 – 59.3	0.6 – 8.5	0.9 – 10.1	2.7 – 50.9	4.5 – 10.0	1.8 – 3.9	0.7 – 0.7	0.1 – 2.4	95 - 523
Mean	338.8	25.9	44.6	63.6	7.0	4.5	10.8	1.62	277.7
SD	884.3	69.4	138.8	162.4	4.8	7.5	31.6	2.53	211.4
±SEM	245.3	19.2	38.5	45.0	1.3	2.1	8.8	0.70	58.6
CV	2.6	2.7	3.1	2.6	0.7	1.7	2.9	1.56	0.8
